# 

**Published:** 1907-07

**Authors:** 


### CONTENTS.

ORIGINAL COMMUNICATIONS—

The Caustive Factor in the Production of Otitis Media in Atlanta. By Claude A. Smith, M. D., President Fulton County Medical Association. Bacteriologist to City of Atlanta ........ 193
The Etiology, Diagnosis and Treatment of Gall Stones. By Willis Jones, M. D.. Atlanta, Ga ........ 197
Discussion of Dr. Jones' Paper ........ 203
State Provision for Inebriates and Drug Habitues. By C. C Stockard, M, D., Atlanta, Ga ........ 205
Mechanical Stimulatiod. By Dr. W. A. Mathews, Hawkinsville, Ga ........ 209
Home Treatment of Tuberculosis. By J. E. Summerfield, M. D., Atlanta. Ga ........ 215
Iodine. By W. C. Abbott, M. D., Chicago, Ill ........ 220
Report of Operation for Multiple Abscess of Pelvis with Slough of Six Inches of Colon; Subsequent Anastamosis with Connell Suture; Recovery. By E C. Davis, M. D,, Professor of Gynecology and Obstetrics, Atlanta School of Medicine, Atlanta. Ga ........ 225
Fulton County Medical Society, May 16th, 1907—Headache- By Wesley Taylor, M. D., Atlanta, Ga ........ 228
Discussion of Dr. Taylor's Paper ........ 230
Pea in the Trachea ........ 232

CORRESPONDENCE ........ 234

EDITORIAL NOTES AND COMMENTS—

A Conundrum ........ 236
One Hundredth Anniversary of the University of Maryland ........ 236
The Value of Sulphur in the Treatment and Prevention of Malaria ........ 237
Palpation, Pus and Proteid ........ 239

NEWS AND NOTES ........ 241
BOOK REVIEWS ........ 244

SELECTIONS AND ABSTRACTS—

Best Method of Anesthetizing Children ........ 246
Is the Doctor a Shylock ? ........ 246
The Transmission of Warts ........ 247
"Misokainia" ........ 247
Infantile Scurvy ........ 248
Germicidal Powers of Vaginal Mucus ........ 248
Eczema as a Danger Signal ........ 249
Treatment of Pneumonia ........ 249
Diet as a Therapeutic Measure in Diseases of the Skin ........ 250
The Use of Heroin ........ 252
Uncontrollable Vomiting of Pregnancy ........ 253
Infantile Diarrhea, With Special Reference to the Use of "Lactated Milk" ........ 255
Bromide Treatment ........ 256

